# Contemporary Land Change Alters Fish Communities in a San Francisco Bay Watershed, California, U.S.A.

**DOI:** 10.1371/journal.pone.0141707

**Published:** 2015-11-18

**Authors:** Kristina Cervantes-Yoshida, Robert A. Leidy, Stephanie M. Carlson

**Affiliations:** 1 Department of Environmental Science, Policy, and Management, University of California, Berkeley, California, United States of America; 2 United States Environmental Protection Agency, San Francisco, California, United States of America; Biodiversity Insitute of Ontario—University of Guelph, CANADA

## Abstract

Urbanization is one of the leading threats to freshwater biodiversity, and urban regions continue to expand globally. Here we examined the relationship between recent urbanization and shifts in stream fish communities. We sampled fishes at 32 sites in the Alameda Creek Watershed, near San Francisco, California, in 1993–1994 and again in 2009, and we quantified univariate and multivariate changes in fish communities between the sampling periods. Sampling sites were classified into those downstream of a rapidly urbanizing area (“urbanized sites”), and those found in less impacted areas (“low-impacted sites”). We calculated the change from non-urban to urban land cover between 1993 and 2009 at two scales for each site (the total watershed and a 3km buffer zone immediately upstream of each site). Neither the mean relative abundance of native fish nor nonnative species richness changed significantly between the survey periods. However, we observed significant changes in fish community composition (as measured by Bray-Curtis dissimilarity) and a decrease in native species richness between the sampling periods at urbanized sites, but not at low-impacted sites. Moreover, the relative abundance of one native cyprinid (*Lavinia symmetricus*) decreased at the urbanized sites but not at low-impacted sites. Increased urbanization was associated with changes in the fish community, and this relationship was strongest at the smaller (3km buffer) scale. Our results suggest that ongoing land change alters fish communities and that contemporary resurveys are an important tool for examining how freshwater taxa are responding to recent environmental change.

## Introduction

The conversion of natural to urban land cover is a considerable threat to species diversity and ecosystem function in freshwater ecosystems [1,2]. Urbanization degrades water quality and alters hydrology, channel structure, and bed composition [3,4], all of which have the potential to impact native biota. Indeed, urbanization has been linked to decreased native species diversity [5,6] as well as the expansion of tolerant and/or nonnative species [7–9]. With continuing human population growth [10] and the anticipated expansion of urban areas in the next few decades [11], there is a pressing need to understand the consequences of contemporary (e.g., 1–2 decades) land change on freshwater biodiversity.

To evaluate the influence of changing land cover on freshwater communities, several approaches have been employed. Most research has focused on single time periods to evaluate how present day community patterns relate to present day land cover (e.g., [12–15]). This approach is useful for elucidating the legacy effects of land use, but provides little insight into the time scale of community shifts. Another approach involves resurveying communities after a given period of time. Historical resurvey studies that span multiple decades have yielded valuable insights into community change in response to land cover change (e.g., [16–20]). Likewise, contemporary resurvey studies following 1–2 decades can be useful for revealing how quickly biotic communities respond to recent land cover change and can provide insight into the dynamics of declining species [21–25].

Beyond considering the influence of recent environmental change on stream biota, effective freshwater conservation also requires identifying the appropriate spatial scale for examining biotic responses to land cover change. Local stream assemblages are influenced by a hierarchy of nested environmental filters that operate at multiple scales from the watershed to the microhabitat scale [26,27]. However, the relative importance of any particular scale on freshwater biota can vary depending of the watershed size, the degree of human disturbance, and other regional differences [28,29]. Similarly, the impacts of land change at different scales and locations within a watershed may have different effects on freshwater communities.

Here, we evaluate the impact of contemporary land change on stream fish communities in a San Francisco Bay watershed in California, U.S.A. through a resurvey study. The fish community was originally surveyed in the mid-1990s (described in [30]), which provided an opportunity to resurvey 32 sites after approximately 16 years to determine how recent urbanization in the region has influenced local fish communities. Our specific objectives were to (1) characterize recent land change within the watershed, (2) characterize fish community change at each sampling site, and (3) explore the influence of land change on fish community change between the two survey periods. We predicted that regions with a greater land cover change would be associated with greater fish community change. We tested this hypothesis using land cover data collected at two scales: a local (3km) buffer immediately upstream of each sampling site and the larger total watershed upstream of each site.

## Materials and Methods

### Study sites

**Fig 1:**
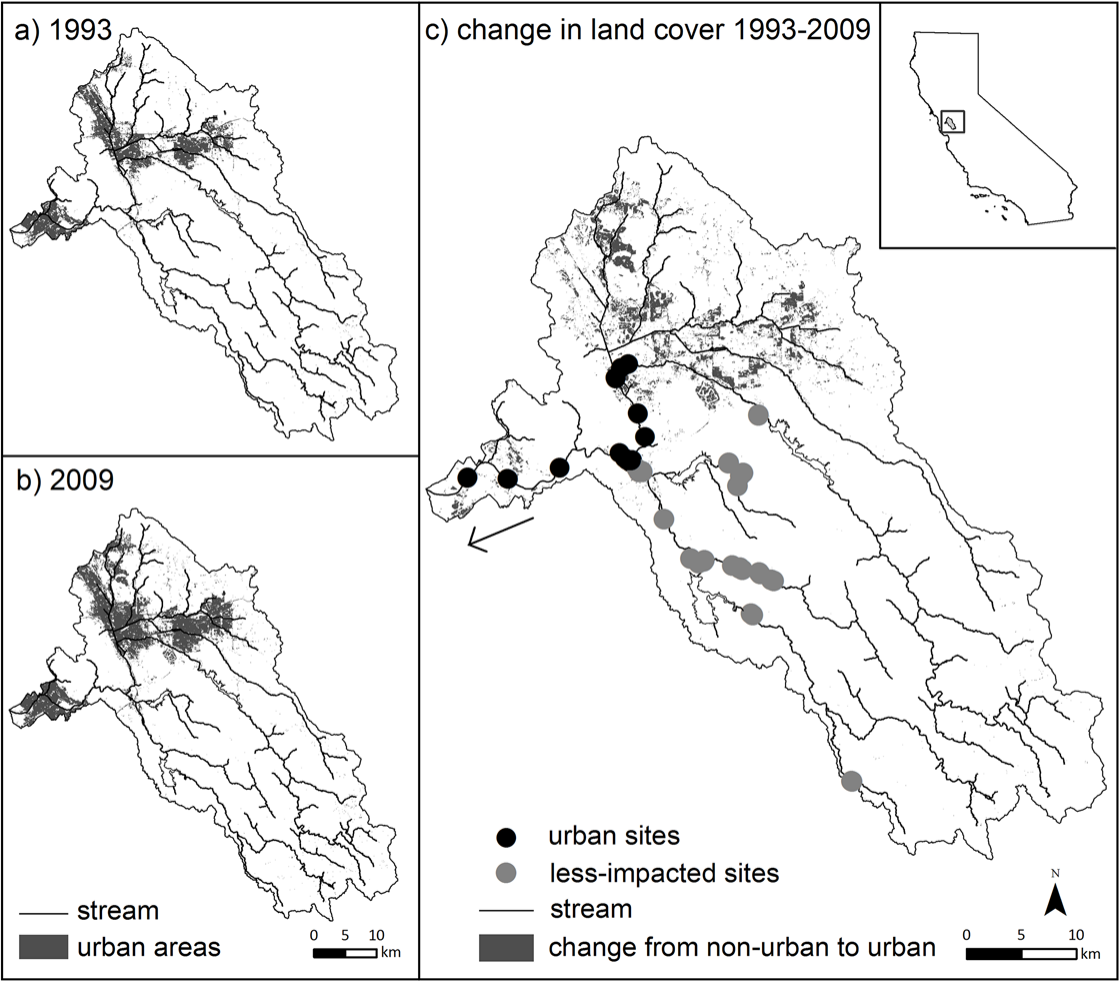
Map of study area showing major streams, sample sites, and the change in land cover to urban areas between 1993 and 2009.

We conducted our study in the Alameda Creek Watershed, the largest watershed of the San Francisco Bay in California, USA (approximately 1800 km^2^), excluding the greater Sacramento and San Joaquin Rivers (Fig 1). This region falls within the Sacramento-San Joaquin Ichthyoprovince, which supports a diverse assemblage of native (n = 40) and nonnative fish species (n = 41) [31]. The Alameda Creek Watershed supports one of the most diverse freshwater fish assemblages within the San Francisco Bay region (approximately 54% of the species found in the greater Sacramento-San Joaquin Ichthyoprovince), including 21 native species and 23 nonnative species at present [32]. This includes two species that are federally threatened (*Oncorhynchus tshawytscha* [Central Valley Fall and Late-Fall ESU] and *Oncorhynchus mykiss* [Central California Coast steelhead DPS]), and five species listed as California Fish Species of Special Concern (*Lavinia exilicauda*, *Lavinia symmetricus*, *Mylopharodon conocephalus*, *Cottus gulosus*, *Archoplites interruptus*) [33].

The Alameda Creek Watershed encompasses multiple land cover types, from upper watershed oak savannas and grasslands, to mid-elevation mixed suburban-low intensity agricultural regions, to low elevation and densely urbanized floodplains. Although much of the upper watershed has remained relatively intact, as much as 78% of the mid- and lower regions of the watershed was converted to agriculture, urban areas, or salt ponds since the early 1880s [32]. The majority of the contemporary land cover change in the watershed has occurred in the Livermore Valley region (see Fig 1). This region includes the city of Dublin, which was recently identified as the third fastest growing city in the state of California [34].

Sample sites were originally chosen to assess the status of native fishes in the watershed, thus sampling was heavily focused on high elevation and less disturbed regions of the watershed. We further restricted our sampling sites to those sites that were wadeable, accessible, and predominately freshwater regions. Sixty-nine sites were initially surveyed by one of us (RAL) between April-October in 1992–1996, with the majority of sites sampled between May -August of 1993 and 1994 [30,35]. In 2009, we resurveyed these sites between May and August, excluding sites that were no longer accessible (n = 1) or were seasonally dry (n = 2). We also excluded sites that were directly upstream and downstream of a small impoundment that was removed as part of a restoration project between the two sampling periods (n = 5). We removed these sites because of concerns that community shifts would be related to the dam removal (e.g., changes in stream depth and width), rather than changes in watershed land use. Finally, some of the earlier sample sites were within a short distance of one another (< 500 m). In these cases, we randomly selected one site to resample, and excluded the other sites (n = 29). After this selection process, 32 sites remained that were sampled in both time periods.

We then classified these 32 sites into one of two categories: (1) those downstream of the developing Livermore Valley (hereafter “urbanized sites”; n sites = 14) and (2) those that were either upstream of Livermore Valley or in adjacent sub-watersheds (hereafter “low-impacted sites”; n sites = 18; Fig 1). These low-impacted sites experienced little land cover change between the two sampling periods (e.g., < 1% change to urban land cover, see Results). This allowed us to compare fish community change in a rapidly urbanizing portion of the watershed to a “control” region that has experienced little land change in recent decades.

### Fish species abundance and distributions

Fish distribution and abundance data were collected by the same lead scientist (RAL) in both the 1990s and 2009. Prior to revisiting sites in 2009, we reviewed the associated data sheets from the mid-1990s for details of site descriptions and sampling methodology. Here we were able to reconstruct an approximately identical study design and effort based on these field notes and from input from RAL. Each site was sampled in same location, and there was no difference in site reach length between the two time periods (paired t-test, *P* = 0.534). At each site, we sampled fishes using single-pass electrofishing in a downstream to upstream direction, using a single-pass to minimize stress on fish during California's summer drought season. For both sampling periods we used shallow riffles or elevational barriers to delimit site boundaries (i.e. to prevent fish escaping upstream from sampled reaches). At deeper sites, we additionally used seine nets to sample the deeper habitat. All captured fish were identified to species, measured for body length (fork length, mm), and released alive at the place of capture. Full details on sampling methodology can be found in [30].

Our fish collection protocol was approved by the University of California Office of Animal Care and Use (Permit # R343), and scientific collection permits specific to this project were obtained from the California Department of Fish and Wildlife. Additionally, we acquired the necessary permits to sample fishes located on land managed by various government agencies (East Bay Regional Park District, San Francisco Public Utilities Commission, Alameda County Flood Control, Livermore Area Recreation and Parks District). No sites in our study were within protected areas, and no listed species were captured or handled.

### Land cover data

We combined Landsat TM Imagery at 30m resolution from July 5, 1993 and June 17, 2009 to produce a stacked multi-temporal land cover change layer. These dates represent the median dates for each sampling period (April-October in 1992–1996 and May-August 2009). We performed a supervised classification using maximum likelihood to create a thematic map representing pairwise land cover change classes that included one change class (natural land cover to urban areas) and two classes that represented no land cover change (i.e., natural land cover and urban areas in both time periods). Although agriculture was present in the watershed, it made up a very small proportion of the total area and did not considerably expand in recent decades. We therefore focused our study on the change from non-urban to urban areas.

Supervised classification training samples were located based on the first author’s knowledge of the region, and on United States Geological Survey (USGS) and United States Department of Agriculture (USDA) high resolution aerial imagery available on Google Earth for June-July of both 1993 and 2009. To assess the accuracy of our land cover change map, we generated 1000 random points to visually compare our derived land cover map with the remote imagery used in the supervised classification. We then calculated standard accuracy metrics including a confusion matrix and kappa statistic [36]. The kappa statistic is a commonly used measurement of accuracy that represents the agreement between the classification and the reference data after removing the proportion of the agreement that might be expected to occur by chance. All land cover classification and accuracy assessments were performed using ERDAS Imagine (Atlanta, USA). Overall we found that the accuracy for our land cover change product was 98.2% and the kappa statistic was 0.93. These values are well within acceptable ranges [37], and are considerably more accurate than commonly used land cover classification data such as the National Land Cover Dataset, which has an accuracy less than 81% for our sample period.

Next we generated individual upstream watershed boundaries for each sample site using USGS 10 m National Elevation Data and hydrology tools in ArcGIS 9.2 [38]. To assess the effect of local land cover, we also delineated a 3km buffer upstream of each site by intersecting a 3km circle with the watershed boundary for each site. The 3km buffer distance was chosen based on the results of Wang et al. [39] who found that imperviousness (i.e., urbanization) within this approximate distance upstream of sampling sites had substantially more influence on stream condition than imperviousness in the total watershed upstream of the site. Next we overlaid the derived land cover change data onto each watershed boundary to determine the degree of land cover change that has occurred upstream of each individual sample site.

### Statistical Analyses

#### Fish community change

To examine fish community change we first characterized the entire fish community by calculating the following univariate metrics for each site: 1) native species richness, 2) nonnative species richness, 3) relative abundance of native individuals (the number of native individuals divided by the total number of individuals across all species captured at a given site), and 4) the relative abundance of each species (the number of individuals of a given species divided by the total number of individuals across all species). We then tested for differences in these metrics between the two time periods using paired t-tests. We performed these analyses separately for the urbanized and low-impacted regions.

Next we followed a standard path of analysis that includes a combination of ordination and multivariate analyses to quantify changes in community composition. First, we used nonmetric multidimensional scaling (NMDS) with Bray-Curtis dissimilarity to visualize differences in community composition between the two sampling periods, again conducting separate analyses for the urbanized and low-impacted sites. Bray-Curtis dissimilarity values range from 0 to 1, where communities that have identical species composition have a value of 0 and communities that share no species have a value of 1. Second, we tested for significant differences in community composition between the two sampling periods using permutation-based analysis of variance (PERMANOVA) with Bray-Curtis dissimilarity [40]. Third, when significant community differences were found, we used indicator-species analysis (ISA; [41]) to identify which species were indicative of a given time period. We considered species with indicator values > 0.50 and *P* < 0.05 as good indicators of a given time period. Fourth, we used a Wilcoxon Signed-Rank test to assess if paired urban sites experienced more community change across time (paired Bray-Curtis dissimilarity values) compared to paired low-impacted sites. Prior to all of the multivariate analyses (NMDS, PERMANOVA, and ISA) we removed species found in only one site, as rare species can obfuscate patterns in community structure. Abundance data were transformed to log(x+1), and all statistical analyses were performed using R [42].

For each of the analyses described above, we included all captured individuals, regardless of size / life stage. Because the abundance and distribution of juveniles can be highly variable across years, it is possible that their inclusion in analyses could generate results that do not reflect long-term shifts in adult populations. To determine whether the inclusion of juveniles strongly influenced our results, we performed a parallel analysis removing all juvenile fish (based on visual inspection of length frequency plots for each species and approximate size thresholds for juveniles described in [31].We found that the multivariate results and the regression analyses were qualitatively similar regardless of whether or not juveniles were included, suggesting that catches of juveniles did not strongly influence the results. Given the qualitative similarity, we present the results of our analyses including all captured fish (i.e., including juveniles).

#### Relationship between the change in land cover and fish communities

We used nonparametric Spearman’s rank correlation to determine the statistical significance and relative strength of correlations (r) between fish community change and land cover change to urban land cover between the two time periods. We used a rank correlation approach because it does not assume linear relationships and our data did not meet the assumptions of normality. To characterize land cover change, we used the percentage of the land cover that changed from non-urban to urban land cover at two scales (within the total watershed and within the 3km buffer area for a particular site). We used the Bray-Curtis distance to describe the pairwise similarity of the fish community composition at a given site between the two sampling periods. Finally, we assessed the relationship between land cover change and the subset of univariate fish measures that differed significantly between the sampling periods, including the change in species richness, the relative abundance of native individuals, and the relative abundance of each species (see Methods above). The changes in univariate fish measurements were assessed by calculating the differences between time periods (i.e., time 2 –time 1), where a negative (positive) value represented a decrease (increase) in the species richness or relative abundance of individuals through time.

## Results

### Land cover change

In both 1993 and 2009, the majority the Alameda Creek Watershed was comprised of natural land cover (grassland and forested areas). Overall the upstream portion of the watershed remained relatively unchanged, whereas contemporary urbanization was concentrated in low elevation reaches and mid-elevation reaches of the Livermore Valley region (Fig 1, Table 1). This was reflected in our results, where the average percent conversion to urban areas was greater among the sites in the “urbanized” region than the “low-impacted” region at both the total watershed (3.8% and < 1%, respectively) and at the 3km buffer scale (3.7% and < 1%, respectively).

**Table 1:** Community and land cover metrics for each sampling period (1990s, 2009) and the change between sampling periods presented separately for low-impacted and urbanized sites. The range for each community metric is noted in parentheses.

		Low-impacted sites			Urbanized sites		
		1990s	2009	Δ	1990s	2009	Δ
community metric	native species richness	2.33 (1–5)	2.00 (1–5)	-0.33	2.86 (2–5)	2.00 (0–5)	-0.86
community metric	nonnative species richness	0.16 (0–3)	0.11 (0–2)	-0.05	0.43 (0–6)	0.86 (0–6)	0.43
community metric	relative abundance of native individuals	98 (89–100)	99 (84–100)	1	90 (31–100)	73 (0–100)	-17
land cover: total watershed	% non-urban	99	99	< 1	94	90	-4
land cover: total watershed	% urban	1	1	< 1	6	10	4
land cover: 3km buffer	% non-urban	99	99	-1	78	75	3
land cover: 3km buffer	% urban	1	1	< 1	22	25	3

All values are based on averages from sites for a given time period and region.

### Fish change

**Fig 2:**
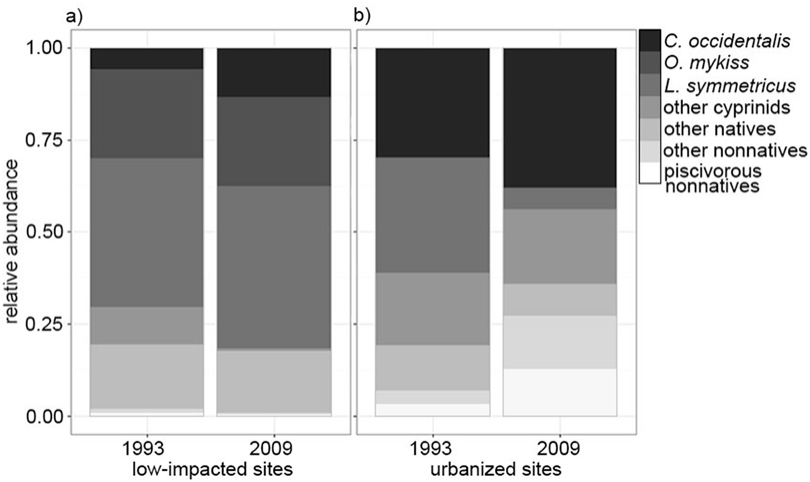
Bar plots of the mean relative abundance for (a) low-impacted sites and (b) urbanized sites for each sample period. Included are shifts in mean relative abundance for three native species: *C*. *occidentalis*, *O*. *mykiss*, and *L*. *symmetricus*. Additionally, we present mean relative abundance for the following groups: other native cyprinids, other natives, other nonnatives, and piscivorous nonnatives (centrarchids and *A*. *flavimanus*). These groups were created for visual purposes and were not used in the analysis.

In total, over 3100 individual fish were captured between the two sample periods, representing 12 families and 23 species (Table 2). Fourteen species were captured in both sample periods, while five species were captured in 2009 that were not captured in the 1990s, and four other species captured in the 1990s that were not captured in 2009. The most widely distributed species during both sample periods was *Lavinia symmetricus* (California roach). *L*. *symmetricus* was also the numerically dominant species in the 1990s, while *Catostomus occidentalis* (Sacramento sucker) was the numerically dominant species in 2009 (Fig 2). Other common species during both time periods included *Oncorhynchus mykiss* (rainbow trout), *Cottus asper* (prickly sculpin), and *Ptychocheilus grandis* (Sacramento pikeminnow; Table 2). The remaining native species and all of the nonnative species comprised a relatively small proportion of the total catch at any given site.

**Table 2:** Information on each species collected, including the proportion of sites where the species was captured and the average relative abundance of a given species across all sites.

			Proportion of sites / Relative abundance of individuals			
Family	Species	NMDS code	1990 slow-impacted	2009 low-impacted	1990s urbanized	2009 urbanized
**Catostomidae**	***Catostomus occidentalis***	COC	0.39/0.06	0.45/0.14	0.79/0.29	0.79/0.40
**Cyprinidae**	***Ptychocheilus grandis***	PGR	0.06/0.02	0.11/0.04	0.79/0.13	0.64/0.20
**Cottidae**	***Cottus asper***	CAS	0.28/0.12	0.56/0.11	0.36/0.05	0.36/0.08
**Cottidae**	***Leptocottus armatus***	N/A	0	0.01/0.01	0.07/0.01	0
**Cyprinidae**	***Lavinia symmetricus***	LSY	0.72/0.43	0.78/0.47	0.86/0.32	0.50/0.06
**Cyprinidae**	***Lavinia exilicauda***	LEX	0.17/0.10	0.11/<0.01	0.43/0.05	0.29/0.01
**Cyprinidae**	***Orthodon microlepidotus***	N/A	0	0	0.07/0.01	0
**Gasterosteidae**	***Gasterosteus aculeatus***	GAC	0.11/0.02	0	0.14/0.06	0.07/0.01
**Petromyzontidae**	***Entosphenus tridentatus***	N/A	0	0.06/<0.01	0	0
**Salmonidae**	***Oncorhynchus mykiss***	OMY	0.61/0.24	0.50/0.25	0	0
Centrarchidae	*Lepomis cyanellus*	LCY	0.17/0.01	0	0.29/0.01	0.21/0.07
Atherinopsidae	*Menidia beryllina*	MBE	0	0	0.14/0.01	0.07/0.01
Centrarchidae	*Micropterus dolomieu*	MDO	0	0	0.07/0.02	0.29/0.05
Centrarchidae	*Lepomis machrochirus*	N/A	0.06/<0.01	0	0.07/<0.01	0
Centrarchidae	*Micropterus salmoides*	MSA	0	0.06/<0.01	0	14/0.01
Cyprinidae	*Cyprinus carpio*	CCA	0.06/<0.01	0	0.14/0.01	0.01/<0.01
Cyprinidae	*Notemigonus crysoleucas*	N/A	0	0	0.07/0.02	0
Cyprinidae	*Carassius auratus*	N/A	0	0	0	0.14/0.04
Fundulidae	*Lucania parva*	N/A	0	0	0.07/<0.01	0.07/0.01
Gobiidae	*Tridentiger bifasciatus*	N/A	0	0	0	0.07/<0.01
Gobiidae	*Acanthogobius flavimus*	N/A	0	0	0	0.07/<0.01
Percidae	*Percina macrolepida*	N/A	0	0	0.07/<0.01	0
Poeciliidae	*Gambusia affinis*	GAF	0.11/0.01	0.06/<0.01	0	0.29/0.08

Species table includes family, species, the labeling code used in the NMDS plot (first letter of the genus followed by the first two letters from the species names), the proportion of sites where a species was captured among all sites for each sample period and site type, and the mean relative abundance of a given species across all sites for each sample period and site type. Native fishes are in bold text and non-native fishes are in plain text. Species found in only one site were excluded from the multivariate analyses (NMDS, PERMANOVA, and ISA) and are denoted with a “N/A” under the NMDS code column.

In low-impacted sites, native species richness did not differ significantly between the 1990s and 2009 (natives: 2.33 and 2.00, respectively; paired t-tests, *P* = 0.33). Native fish comprised the vast majority of the catch at low-impacted sites during both time periods, where the average relative abundance of native individuals at each site was 98% and 99% for the mid-1990s and 2009, respectively (Table 1), a difference that was non-significant (paired t-test, *P* = 0.34). The two dominant species during both time periods at low-impacted sites were *L*. *symmetricus* and *O*. *mykiss* (Fig 2A), and there were no significant differences in the relative abundance of any native species between the two time periods (paired t-test, *P* > 0.05). Non-native species richness was low overall in low-impacted sites, and also did not differ significantly between time periods (0.16 and 0.11, respectively, *P* = 0.29).

At urbanized sites, there was a significant decrease in the richness of native species between the 1990s and 2009 (2.86 and 2.00, respectively, paired t-test, *P* = 0.002; Table 1). The numerically dominant native species in urbanized sites in the 1990s was *L*. *symmetricus* (Fig 2B), but the relative abundance of *L*. *symmetricus* was significantly lower in 2009 than in the 1990s (paired t-test, *P* = 0.004). As a consequence, *C*. *occidentalis* emerged as the numerically dominant species in 2009 for urbanized sites (Fig 2B). No other univariate community metrics differed significantly between the two time periods for urbanized sites (e.g., relative abundance of individual native species, non-native species richness; *P* > 0.05).

**Fig 3:**
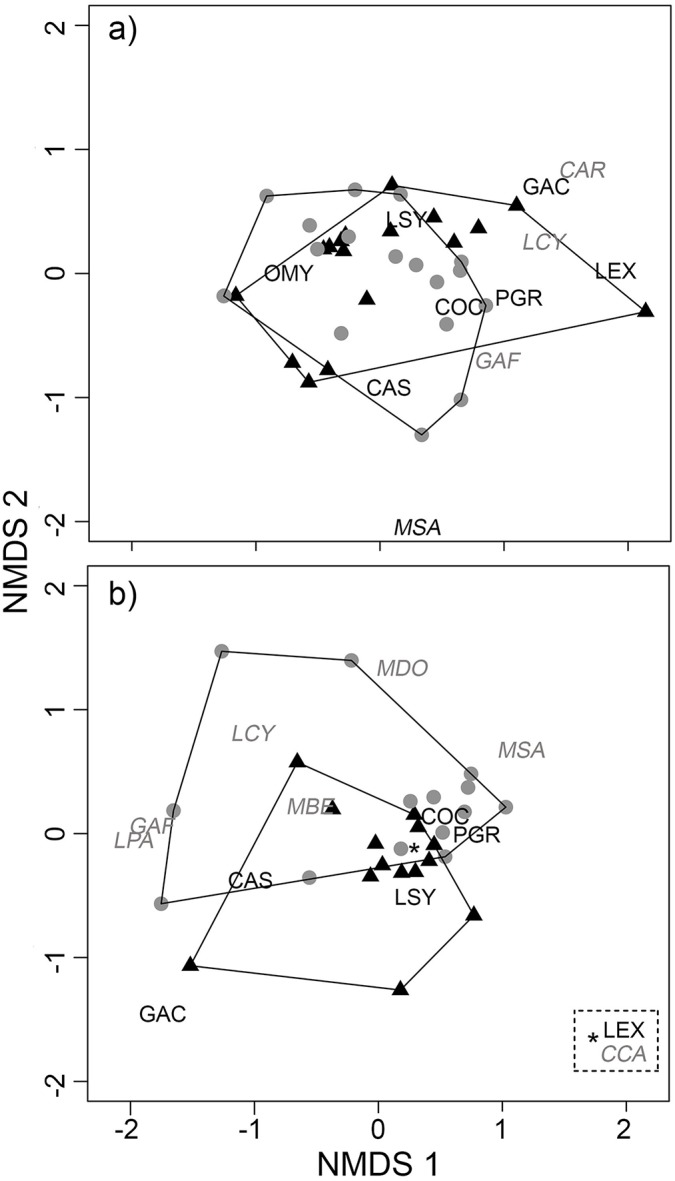
Nonmetric multidimensional plot of species abundance for (a) low-impacted sites and (b) urbanized sites. Species labels match the codes provided in Table 2. Native species are shown in plain black font, and nonnative species are shown in gray italicized font. Sites sampled in the 1990s are denoted with black triangles and sites sampled in 2009 are denoted with gray circles. Sites located close together in plot space represent sites with more similar species assemblages. The convex hulls enclose sites sampled in each time period (1990s, 2009).

**Fig 4:**
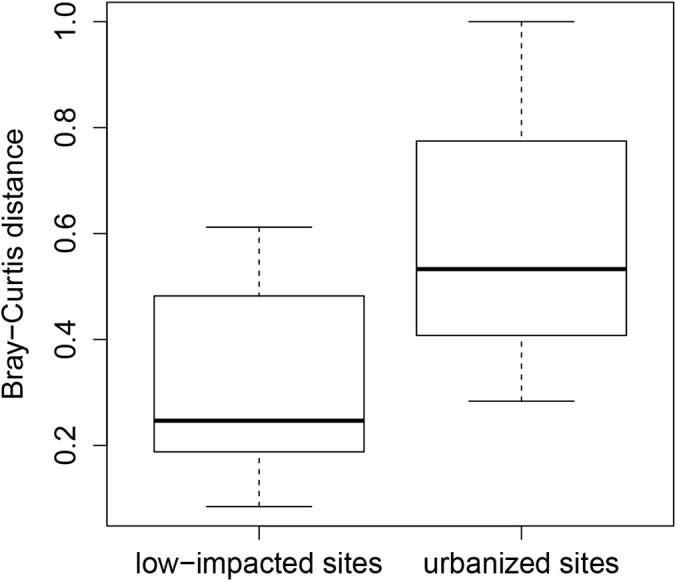
Boxplots comparing Bray-Curtis dissimilarity distances for sites sampled in both time periods, presented separately for low-impacted sites and urbanized sites.

For our multivariate analyses (NMDS, PERMANOVA, and ISA), we excluded three native species and seven nonnative species that were captured in only one time period and in low abundance (Table 2, labeled “N/A” under NMDS code). We therefore included a total of 2945 individual fish representing seven native and six non-native species for our multivariate analyses. These analyses revealed that the fish community composition did not change at the low-impacted sites between the two sample periods (_PERMANOVA_, pseudo-F_1,33_ = 0.44, *P* = 0.65). Indeed, the NMDS plot (stress = 0.10), with convex hulls enclosing sites for a given sample period, revealed considerable overlap of fish communities during the two time periods for low-impacted sites (Fig 3A). In contrast, fish community composition at the urbanized sites changed significantly between the two sample periods (_PERMANOVA,_ pseudo-F_1,26_ = 2.2, *P* = 0.04). The NMDS plot of urbanized sites for each time period suggested a directional change of the communities between sampling periods, with nonnative species and native *C*. *occidentalis* more strongly associated with 2009 samples and native *L*. *symmetricus* more strongly associated with 1990s samples (stress = 0.12; Fig 3B). *L*. *symmetricus* emerged as a significant indicator species for our urbanized sites in the 1990s (Indicator value = 0.60, *P* = 0.03). In contrast, no significant indicator species were found for urbanized sites in 2009. When comparing low-impacted and urban sites, we found that fish communities in urban sites differed more between the two time periods than those in low-impacted sites (mean Bray-Curtis dissimilarity values = 0.60 and 0.33, respectively, Wilcoxon Rank test, p = 0.002; Fig 4).

### Relationship between the changes in land cover and fish communities

**Fig 5:**
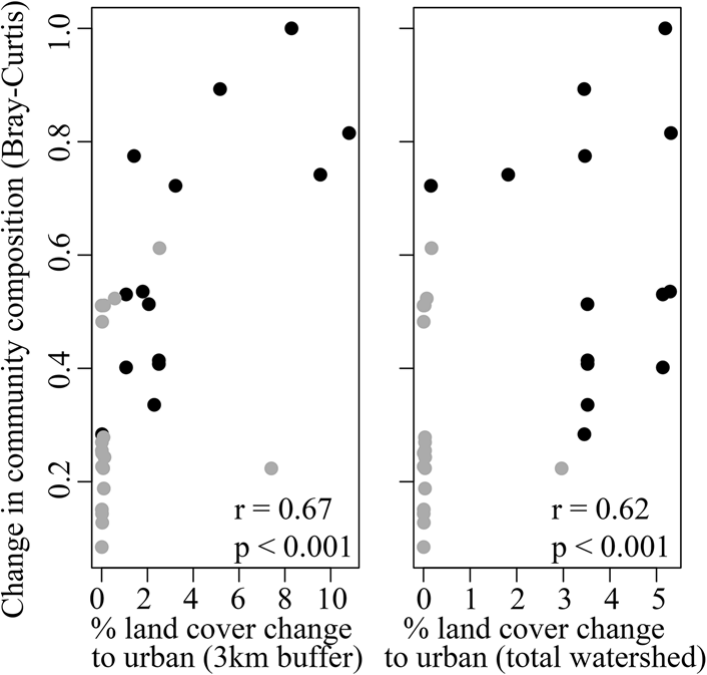
Change in community composition versus change in land cover. Plotted is the relationship between the change in community composition using Bray-Curtis dissimilarity and the change from non-urban to urban land cover at two scales: (a) within 3km upstream of the site, and (b) the total watershed area upstream of the site. Bray-Curtis values closer to one indicate that communities have a high degree of dissimilarity. Black circles represent urbanized sites and gray circles represent low-impacted sites. Spearman's rank correlation coefficient (r) and level of significance (p) are indicated on each panel.

**Fig 6:**
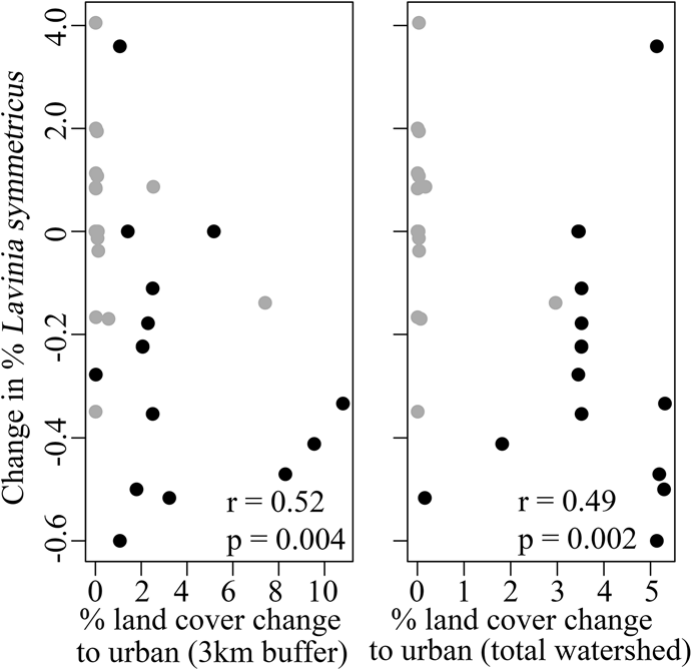
Change in the relative abundance of *Lavinia symmetricus* versus change in land cover. Plotted is the relationship between the change in the relative abundance of *L*. *symmetricus* (California roach) with the change from non-urban to urban land cover at two scales: (a) within 3km upstream of the site, and (b) the total watershed area upstream of the site. For both plots, black circles represent urbanized sites and gray circles represent low-impacted sites. Spearman's rank correlation coefficient (r) and level of significance (p) are indicated on each panel.

To assess the relationship between the change in land cover and fish communities, we focused on the fish community metrics that differed significantly between the two sample periods. The change in fish community composition between sites sampled in both the 1990s and 2009 (measured as Bray-Curtis dissimilarity) was positively related to the change from non-urban to urban land cover. This relationship was significant at both scales, though it was stronger at the local 3km scale (r = 0.67, p < 0.001; Fig 5A) compared to the total watershed scale (r = 0.62, p < 0.001; Fig 5B). Overall, sites in the urbanized portion of the watershed experienced the highest land cover change and the largest change in fish community composition. Additionally we found that the relative abundance of *L*. *symmetricus* was negatively related to land cover change, a result that was consistent at both scales (3km watershed, r = -0.52, p = 0.004, total watershed r = -0.49, p = 0.002; Fig 6A and 6B). We did not find a significant relationship between native species richness and land cover change at either scale.

## Discussion

Our results showed that contemporary urbanization—over an approximately 16 year period—in the 1800 km^2^ Alameda Creek Watershed was associated with fish community change. Importantly, we found significant changes in fish communities only in those portions of the watershed that had experienced recent urbanization, while no such changes were detected in regions that experienced little land cover change during this same time period. The relationship between land cover change and fish community change was the strongest at the local scale (i.e., 3km buffer zone upstream of a site) as opposed to the land change in the total watershed upstream of our sampling sites.

### Fish community changes

While our study focused on contemporary change, earlier research in the study watershed provides a longer scale perspective on fish community change due to human impacts. Leidy (2007) compiled a time series of fish extirpations and introductions in the Alameda Creek Watershed from 1855–2012 using diverse data sources, including direct sampling, historical literature, and museum specimens (Table 3; [30,32]). The results show that four fish species have been extirpated from this watershed, including one that is now globally extinct (*Gila crassicauda*, thicktail chub). Two anadromous species were extirpated from the system, likely due to the construction of three major dams (between 1925 and 1968) which blocked access to upstream areas. The extirpation of *Pogonichthys macrolepidotus* (Sacramento splittail), an estuarine fish, may have occurred following the draining of a major wetland and the degradation of tidal wetlands in the lower reaches of the watershed [32]. In contrast, nonnatives have been steadily increasing through time in this watershed, with nonnative richness nearly doubling from 1953–1969 to the present day (Table 3).

**Table 3:** Historical changes in fish fauna of the Alameda Creek Watershed 1865–2012 [30,32].

Scientific name	Period of Record					Current status
	1855–1860	1895–1948	1953–1969	1972–1987	1992–2012	
***Gila crassicauda***	X					E
***Pogonichthys macrolepidotus***	X	P	X			E
***Oncorhynchus kisutch***	P	P	X			E
***Oncorhynchus tshawytscha***	X	U	U	U	X	R
***Rhinichthys osculus***	P	X	U	U	U	U
***Cottus gulosus***	P	X	U	U	X	U
***Mylopharodon conocephalus***	P	X	X	P	X	R
***Hysterocarpus traski***	X	X	P	X	X	R^1^
***Cymatogaster aggregata***	P	P	P	X	X	I^2^
***Lampetra ayresii***	P	P	X	X	X	I
***Lampetra cf*. *pacifica***	P	P	P	P	X	C
***Entosphenus tridentatus***	X	X	X	X	X	C
***Lavinia exilicauda***	P	X	X	X	X	C
***Lavinia symmetricus***	X	X	X	X	X	C
***Orthodon microlepidotus***	P	X	X	X	X	I
***Ptychocheilus grandis***	P	X	X	X	X	C
***Catostomus occidentalis***	X	X	X	X	X	C
***Oncorhynchus mykiss***	P	X	X	X	X	C
***Gasterosteus aculeatus***	X	X	X	X	X	C
***Archoplites interruptus***	X	X	X	X	X	R^1^
***Cottus asper***	X	X	X	X	X	C
***Leptocottus armatus***	P	P	P	X	X	C
***Gillichthys mirabilis***	P	P	P	X	X	U^2^
***Platichthys stellatus***	P	P	P	P	X	I^3^
*Micropterus dolomieu*		X	X	X	X	C
*Salmo trutta*		X	X			E
*Cyprinus carpio*			X	X	X	C
*Ameiurus catus*			X	X	X	R^3^
*Ameiurus nebulosus*			X	X	X	R^3^
*Gambusia affinis*			X	X	X	C
*Pomoxis nigromaculatus*			X	X	X	R^3^
*Lepomis cyanellus*			X	X	X	C
*Lepomis macrochirus*			X	X	X	C
*Micropterus salmoides*			X	X	X	C
*Carassius auratus*			X	X	X	C
*Notemigonus crysoleucas*			X	X	X	C
*Lucania parva*			X	X	X	C
*Dorosoma petenense*				X	X	R^3^
*Ictalurus punctatus*				X	X	R^3^
*Ameiurus melas*				X	X	R^3^
*Menidia beryllina*				X	X	C
*Morone saxatilis*				X	X	R^3^
*Lepomis microlophus*				X	X	R^3^
*Percina macrolepida*				X	X	R
*Acanthogobius flavimanus*				P	X	C^2^
*Micropterus coosae*					X	R^3^
*Gila bicolor*					X	E
*Tridentiger bifasciatus*					X	I^3^

Native fishes are in bold text and non-native fishes are in plain text. Criteria were developed to assess the reliability of the data, where X = definite occurrences determined by observations made from stream surveys, published and unpublished literature and reports, and museum collections (see [43] for more details). P = occurrences that are not recorded but are likely present, and U = species with unknown status. Contemporary presence data were determined from multiple sources (e.g., personal observations, surveys from local agencies) and not just from data used for this study. The estimate of the current status of species are given, where E = extirpated/extinct, R = rare, I = intermediate, C = common, U = status unknown. Superscripts denote habitat type other than streams that species are primarily found, where ^1^ = off-channel ponds, ^2^ = tidal reaches, and ^3^ = reservoirs.

Our examination of contemporary fish community change provided little evidence of new introductions or extirpations between the mid-1990s and 2009, although there were nine species that were found during one time period but not the other. These nine species were rare and encountered at just a few sampling sites. Moreover, all of these species have been detected in other recent surveys by the authors in different locations within the watershed, although in low abundances and with limited distributions. That there have been no contemporary extirpations from the watershed may reflect the fact that the highly sensitive species with restricted ranges and low abundances historically were already extirpated from the watershed prior to our study (e.g., *O*. *kisutch*, Table 3). Another possible explanation is that the extant species represent a subset of fairly resilient species that are able to persist in areas of moderate human disturbance or remain in undisturbed habitat in the headwaters [35].

Although we did not detect any recent extirpations, our analyses revealed a decrease in average native species richness and shifts in the dominance of extant species. Shifts in the species richness of depauperate communities such as ours, where headwater communities are often comprised of fewer than three species, could lead to large changes in the Bray-Curtis dissimilarity index. We however found no statistical difference in the mean Bray-Curtis values when species were added or removed between sites with fewer than three species and sites with greater than three species (t-test, *P* = 0.15), suggesting that low species richness at our headwater (and low-impacted) sites did not unduly influence our results.

Overall, we found that the changes in community composition occurred at sites experiencing recent urbanization, but not at sites with little recent land change. Specifically, we found that the distribution of the native *Lavinia symmetricus* (California roach) decreased over time in urbanized sites (detected at 86% of sites in the 1990s but only 50% of sites in 2009). This result was not surprising because common species are often the first species within communities to be affected by environmental perturbations [44]. Although *L*. *symmetricus* is widespread in streams of the region, they are vulnerable to human disturbances and nonnative predators. In fact, they are often absent from sites invaded by nonnative *Lepomis cyanellus* [31], which is a cause for concern since this and other nonnative centrarchid species were captured at more sites in 2009 than in the 1990s.

Other studies have shown that native cyprinids can be replaced by other wide spread species following environmental perturbation [18]. Here we found that native *Catostomus occidentalis* (Sacramento sucker) emerged as the numerically dominant fish species at sites experiencing recent urbanization. While many of the *C*. *occidentalis* captured in 2009 were juveniles, excluding juveniles from our analyses did not qualitatively change our results or conclusions (see Methods). Moreover, *C*. *occidentalis* can tolerate a range of water quality conditions, and unlike many native cyprinids with which they co-occur, they are commonly found in waters dominated by nonnatives [31]. This ability to persist in altered habitats is apparent in the results of our study, and further increases in their relative abundance might be expected as urbanization increases—to a point—over time. Both the decrease in *L*. *symmetricus* and the increase in *C*. *occidentalis* may have broader impacts for the ecosystem and food web, as common species such as these are often are involved in many biotic interactions [44] and can have cascading effects on other organisms.

While long-term historical resurveys commonly report extirpations or range shifts [45,46], contemporary resurveys across shorter time periods are important for detecting subtle shifts in communities. For example, a survey of aquatic macroinvertebrates in Central Australia across a contemporary 12-year period showed slight shifts in species richness following major disturbances in the watershed [47]. Likewise, Heard et al. [48] found shifts in the ratio of native and nonnatives species in shoreline plant communities across a contemporary 10-year period. Contemporary resurvey studies are also useful in identifying patterns in community trajectories. For example, a recent study spanning a 27-year period found that fish communities followed a directional trajectory with a return to a former state following various flow disturbances [25]. The subtle shifts in the community dynamics that we detected in our study may also be part of a long-term directional trajectory and may foretell future conservation challenges, which could help identify species in decline prior to extirpation. For example, our results suggest a decline in a tolerant native species in rapidly urbanizing reaches. Moreover, some native species in our study were encountered at too few sites to be included in analyses (e.g., Pacific lamprey, *Entosphenus tridentatus*), while other rare species were not captured in our surveys despite being known to occur in the watershed (e.g., hard head, *Mylopharodon conocephalus*; [49]). Monitoring rare species such as these require additional effort because of issues with detection, but such efforts are clearly needed to monitor their status and to identify threats to their viability.

### Influence of scale

We found that fish community shifts were more strongly associated with local land cover change (within a 3km buffer) than changes at the scale of the total watershed upstream of a site. This result may be a consequence of the spatial pattern of urbanization within our study watershed, i.e., it is concentrated in the mid and lower regions. For example, when focusing on the urbanized sites, the influence of local land change (e.g., degraded water quality, excess nutrients, altered hydrology) was possibly diluted when considering the larger watershed scale, where–in this system–most of the upper watershed has experienced little land change. Similarly, urban and agricultural development in the upper watershed also may have been dampened by the relatively intact riparian areas in the upper watershed that may, for example, allow for the infiltration of pollutants. Other studies have also shown that regional factors such as watershed size [50], the degree of human disturbance [28,29], and the configuration of land cover change within the watershed [51] are important factors influencing stream fishes and macroinvertebrates, and may determine the dominant scale of influence in a particular system. This suggests that the dominant scale of influence of land change may be context specific, thus highlighting the need for regionally-based studies to fully understand how land cover change influences freshwater biota.

The relationship between land cover change and changes in freshwater fishes may also vary across time. For example, there may be delayed responses in the recovery of a stream system following periods of land cover change. Henshaw and Booth [52] found that stream banks restabilized in one to two decades following a period of active urbanization in the watershed. Moreover, certain land use practices may also have legacy effects on stream systems. Harding et al. [16] showed that the diversity of freshwater macroinvertebrates and fishes had a stronger relationship with agricultural land cover from forty years prior compared to recent land cover change, despite recent restoration efforts in the watershed. There may also be lags between land cover change and the subsequent effects on stream biota, and these lag periods may vary among different stressors. For example, dissolved pollutants, such as nutrients, can quickly disperse downstream, while sediment and attached pollutants may take many years to move downstream [53]. Similarly, pollutants that are carried through groundwater may move slower than those that move through surface water flow.

Lag time responses from watershed perturbations may also vary among species with different life histories, behaviors, and physiology tolerances [54]. For example, there may be a delay in the extirpation of long-lived species when the perturbation affects reproduction or juvenile survival. Together these physical and biotic lags may lead to extinction debts to be paid in this system, where species may be lost in the future as a result of recent land use changes [55]. Consequently, while contemporary resurveys at short intervals may help to identify transitional changes in community structure that historical surveys might overlook, it is possible that shorter sampling intervals may miss more drastic changes in the fish community that have yet to occur. Beyond accounting for lagged effects, it is worth mentioning that it is challenging to relate changes in the watershed to changes in fish communities when the time since urbanization has likely differed among sites. For our study we focused on land use change that was concentrated in time (i.e., within approximately 1.5 decades) to avoid large differences in time since disturbance among our sites. Resurveys following varying time periods may help reveal both the lagged effects and transitional changes in freshwater fish communities following land conversion and other watershed perturbations.

### Management implications

As freshwater fish populations continue to decline both locally (e.g., [49]) and globally [56], research exploring causes of decline are needed to guide conservation and management efforts. Urbanization is considered one of the leading factors influencing freshwater biodiversity [2], and urban areas are expected to expand significantly across the globe in the coming decades [57]. Between 1993 and 2009, the percent of urban land cover in our focal watershed increased from 7% to 10%, but it was higher for many of our sample sites within the rapidly urbanizing region of the watershed (e.g., from 8% to nearly 14%). Previous research has shown that even a relatively low percentage of urbanization in a watershed can have a disproportionately large effect on stream macroinvetebrates and fishes. King et al. [58] found that 80% of stream macroinvertebrates declined in watersheds with as little as 0.5–2% urbanization. Likewise, a meta-analysis on land cover change reported an approximate 6% loss of aquatic species richness for 10% loss of natural land cover based on data collected from multiple systems [59]. Hence, if the current rate of land cover change in Alameda Creek Watershed continues, future declines or even extirpations are likely to occur.

In our study region and in many others, undeveloped regions were primarily located in the headwater reaches. Reservoirs, other migration barriers, and the encroachment of suburban development may result in the fragmentation of watersheds and reduced connectivity to these upper headwater regions, which can be important refugia for many native fishes. Headwater streams in the San Francisco Bay region support several endemic and threatened fishes, in addition to one native fish (*L*. *symmetricus*) that has declined in the lower elevation and urbanizing portions of the watershed. As such, our results support a conservation strategy of protecting existing undeveloped regions and the surrounding low- to mid- developed regions in urbanizing watersheds. Overall our study suggests that contemporary land change is associated with subtle changes in fish communities that may foretell future declines, and highlights the need for additional surveys to understand the longer-term effects of recent land change on fish communities.

## References

[1] McDonald RI, Marcotullio PJ, Güneralp B. Urbanization and global trends in biodiversity and ecosystem services In: Elmqvist T, Fragkias M, Goodness J, Güneralp B, Marcotullio PJ, McDonald RI, et al, editors. Urbanization, Biodiversity and Ecosystem Services: Challenges and Opportunities. Springer; 2013 pp. 31–52.

[2] Sala OE. Global biodiversity scenarios for the year 2100. Science. 2000;287: 1770–1774. 10.1126/science.287.5459.1770 10710299

[3] Paul MJ, Meyer JL. Streams in the urban landscape. Annu Rev Ecol Syst. 2001;32: 333–365.

[4] Walsh CJ, Roy AH, Feminella JW, Cottingham PD, Groffman PM, Morgan RP II. The urban stream syndrome: current knowledge and the search for a cure. J North Am Benthol Soc. 2005;24: 706–723.

[5] Riley SPD, Busteed GT, Kats LB, Vandergon TL, Lee LFS, Dagit RG, et al Effects of urbanization on the distribution and abundance of amphibians and invasive species in Southern California streams. Conserv Biol. 2005;19: 1894–1907. 10.1111/j.1523-1739.2005.00295.x

[6] Helms BS, Schoonover JE, Feminella JW. Seasonal variability of landuse impacts on macroinvertebrate assemblages in streams of western Georgia, USA. J North Am Benthol Soc. 2009;28: 991–1006. 10.1899/08-162.1

[7] Morgan RP, Cushman SF. Urbanization effects on stream fish assemblages in Maryland, USA. J North Am Benthol Soc. 2005;24: 643–655.

[8] Roy AH, Freeman MC, Freeman BJ, Wenger SJ, Ensign WE, Meyer JL. Investigating hydrologic alteration as a mechanism of fish assemblage shifts in urbanizing streams. J North Am Benthol Soc. 2005;24: 656–678.

[9] Marchetti MP, Lockwood JL, Light T. Effects of urbanization on California’s fish diversity: differentiation, homogenization and the influence of spatial scale. Biol Conserv. 2006;127: 310–318. 10.1016/j.biocon.2005.04.025

[10] Gerland P, Raftery AE, Sevcikova H, Li N, Gu D, Spoorenber T, et al World population stabilization unlikely this century. Science. 2014;346: 234–237. 10.1126/science.1257469 25301627 PMC4230924

[11] Seto KC, Fragkias M, Güneralp B, Reilly MK. A meta-analysis of global urban land expansion. PLoS One. 2011;6: e23777 10.1371/journal.pone.0023777 21876770 PMC3158103

[12] Lammert M, Allan JD. Assessing biotic integrity of streams: effects of scale in measuring the influence of land use/cover and habitat structure on fish and macroinvertebrates. Environ Manage. 1999;23: 257–270. 9852191 10.1007/s002679900184

[13] Fitzpatrick FA, Scudder BC, Lenz BN, Sullivan DJ. Effects of multi-scale environmental characteristics on agricultural stream biota in Eastern Wisconsin. J Am Water Resour Assoc. 2001;37: 1489–1507.

[14] De Jesús-Crespo R, Ramírez A. Effects of urbanization on stream physicochemistry and macroinvertebrate assemblages in a tropical urban watershed in Puerto Rico. J North Am Benthol Soc. 2011;30: 739–750. 10.1899/10-081.1

[15] Sály P, Takács P, Kiss I, Bíró P, Eros T. The relative influence of spatial context and catchment- and site-scale environmental factors on stream fish assemblages in a human-modified landscape. Ecol Freshw Fish. 2011;20: 251–262. 10.1111/j.1600-0633.2011.00490.x

[16] Harding JS, Benfield EF, Bolstad PV, Helfman GS, Jones EB III. Stream biodiversity: the ghost of land use past. Proc Natl Acad Sci U S A. 1998;95: 14843–14847. 9843977 10.1073/pnas.95.25.14843 PMC24537

[17] Wenger SJ, Peterson JT, Freeman MC, Freeman BJ, Homans DD. Stream fish occurrence in response to impervious cover, historic land use, and hydrogeomorphic factors. Can J Fish Aquat Sci. 2008;65: 1250–1264. 10.1139/F08-046

[18] Johnston CE, MacEina MJ. Fish assemblage shifts and species declines in Alabama, USA streams. Ecol Freshw Fish. 2009;18: 33–40. 10.1111/j.1600-0633.2008.00319.x

[19] Maloney KO, Weller DE. Anthropogenic disturbance and streams: land use and land-use change affect stream ecosystems via multiple pathways. Freshw Biol. 2011;56: 611–626. 10.1111/j.1365-2427.2010.02522.x

[20] Johnson PTJ, McKenzie VJ, Peterson AC, Kerby JL, Brown J, Blaustein AR, et al Regional decline of an iconic amphibian associated with elevation, land-use change, and invasive species. Conserv Biol. 2011;25: 556–566. 10.1111/j.1523-1739.2010.01645.x 21342266

[21] Patton TM, Rahel FJ, Hubert WA. Using historical data to assess changes in Wyoming’s fish fauna. Conserv Biol. 1998;12: 1120–1128.

[22] Poole K, Downing J. Relationship of declining mussel biodiversity to stream-reach and watershed characteristics in an agricultural landscape. J North Am Benthol Soc. 2004;23: 114–125.

[23] Gibbs J, Whiteleather K, Schueler F. Changes in frog and toad populations over 30 years in New York State. Ecol Appl. 2005;15: 1148–1157.

[24] Labay B, Cohen AE, Sissel B, Hendrickson DA, Martin DF, Sarkar S. Assessing historical fish community composition using surveys, historical collection data, and species distribution models. PLoS One. 2011;6: e25145 10.1371/journal.pone.0025145 21966438 PMC3178614

[25] Matthews WJ, Marsh-Matthews E, Cashner RC, Gelwick F. Disturbance and trajectory of change in a stream fish community over four decades. Oecologia. 2013;173: 955–69. 10.1007/s00442-013-2646-3 23543217

[26] Frissell CA, Liss WJ, Warren CE, Hurley MD. A hierarchical framework for stream habitat classification: viewing streams in a watershed context. Environ Manage. 1986;10: 199–214. 10.1007/BF01867358

[27] Poff NL. Stream ecology landscape filters and species traits: towards mechanistic understanding and prediction in stream ecology. J North Am Benthol Soc. 1997;16: 391–409.

[28] Strayer DL, Beighley RE, Thompson LC, Brooks S, Nilsson C, Pinay G, et al Effects of land cover on stream ecosystems: roles of empirical models and scaling issues. Ecosystems. 2003;6: 407–423. 10.1007/s10021-002-0170-0

[29] Stanfield L, Kilgour B. How proximity of land use affects stream fish and habitat. River Res Appl. 2012;905: 891–905. 10.1002/rra

[30] Leidy RA. Ecology, assemblage structure, distribution, and status of fishes in streams tributary to the San Francisco Estuary, California. San Fr Estuary Inst Contrib No 530 Oakland, CA. 2007;

[31] Moyle PB. Inland fishes of California: revised and expanded 2nd ed. Berkeley, CA: University of California Press; 2002.

[32] Stanford B, Grossinger RM, Beagle J, Askevold RA, Leidy RA, Beller EE, et al. Alameda Creek Watershed historical ecology study, SFEI Publication #679. Richmond, CA: San Francisco Estuary Institute; 2013.

[33] Moyle PB, Quiñones RM, Katz JV, Weaver J. Fish Species of Special Concern in California [Internet]. Sacramento; 2015. Available: www.wildlife.ca.gov

[34] Gutierrez M. Bay Area is fastest-growing region in state. San Francisco Gate. 1 May 2014.

[35] Leidy RA, Cervantes-Yoshida K, Carlson SM. Persistence of native fishes in small streams of the urbanized San Francisco Estuary, California: acknowledging the role of urban streams in native fish conservation. Aquat Conserv Mar Freshw Ecosyst. 2011;21: 472–483. 10.1002/aqc.1208

[36] Congalton RG. A review of assessing the accuracy of classifications of remotely sensed data. Remote Sens Environ. 1991;37: 35–46. 10.1016/0034-4257(91)90048-B

[37] Foody GM. Status of land cover classification accuracy assessment. Remote Sens Environ. 2002;80: 185–201. 10.1016/S0034-4257(01)00295-4

[38] ESRI: Environmental Systems Resource Institute. ArcMap 9.2, Redlands, CA. Redlands, CA; 2009.

[39] Wang L, Lyons J, Kanehl P, Bannerman R. Impacts of urbanization on stream habitat and fish across multiple spatial scales. Environ Manage. 2001;28: 255–266. 10.1007/s002670010222 11443388

[40] Anderson MJ. A new method for non-parametric multivariate analysis of variance. Austral Ecol. 2001;26: 32–46.

[41] Dufrene M, Legendre P. Species assemblages and indicator species: the need for a flexible asymmetrical approach. Ecol Monogr. 1997;67: 345–366.

[42] R Core Team. R: A language and environment for statistical computing [Internet] Vienna, Austria: R Foundation for Statistical Computing; 2014 Available: http://www.r-project.org/

[43] Leidy R, Becker G, Harvey B. Historical status of coho salmon in streams of the urbanized San Francisco estuary, California. Calif Fish Game. 2005;91: 219–254.

[44] Gaston KJ. Valuing common species. Science. 2010;327: 154–155. 10.1126/science.1182818 20056880

[45] Tingley MW, Beissinger SR. Detecting range shifts from historical species occurrences: new perspectives on old data. Trends Ecol Evol. 2009;24: 625–33. 10.1016/j.tree.2009.05.009 19683829

[46] Magurran AE, Baillie SR, Buckland ST, Dick JM, Elston DA, Scott EM, et al Long-term datasets in biodiversity research and monitoring: assessing change in ecological communities through time. Trends Ecol Evol. 2010;25: 574–82. 10.1016/j.tree.2010.06.016 20656371

[47] Brim-Box J, Davis J, Strehlow K, McBurnie G, Duguid A, Brock C, et al Persistence of central Australian aquatic invertebrate communities. Mar Freshw Res. 2014;65: 562–572. 10.1071/MF13131

[48] Heard MJ, Sax DF, Bruno JF. Dominance of non-native species increases over time in a historically invaded strandline community. Divers Distrib. 2012;18: 1232–1242. 10.1111/j.1472-4642.2012.00918.x

[49] Moyle PB, Katz JVE, Quiñones RM. Rapid decline of California’s native inland fishes: a status assessment. Biol Conserv. 2011;144: 2414–2423.

[50] Esselman PC, Allan JD. Relative influences of catchment- and reach-scale abiotic factors on freshwater fish communities in rivers of northeastern Mesoamerica. Ecol Freshw Fish. 2010;19: 439–454. 10.1111/j.1600-0633.2010.00430.x

[51] Kearns FR, Kelly NM, Carter JL, Resh VH. A method for the use of landscape metrics in freshwater research and management. Landsc Ecol. 2005;20: 113–125. 10.1007/s10980-004-2261-0

[52] Henshaw PC, Booth DB. Natural restabilization of stream channels in urban watersheds. J Am Water Resour Assoc. 2001;36: 1219–1236.

[53] Meals DW, Dressing SA, Davenport TE. Lag time in water quality response to best management practices: a review. J Environ Qual. 2010;39: 85–96. 10.2134/jeq2009.0108 20048296

[54] Kuussaari M, Bommarco R, Heikkinen RK, Helm A, Krauss J, Lindborg R, et al Extinction debt: a challenge for biodiversity conservation. Trends Ecol Evol. 2009;24: 564–571. 10.1016/j.tree.2009.04.011 19665254

[55] Tilman D, May RM, Lehman CL, Nowak MA. Habitat destruction and the extinction debt. Nature. 1994;371: 65–66. 10.1038/371065a0

[56] Burkhead NM. Extinction rates in North American freshwater fishes, 1900–2010. Bioscience. 2012;62: 798–808.

[57] Angel S, Parent J, Civco DL, Blei A, Potere D. The dimensions of global urban expansion: estimates and projections for all countries, 2000–2050. Prog Plann. 2011;75: 53–107. 10.1016/j.progress.2011.04.001

[58] King RS, Baker ME, Kazyak PF, Weller DE. How novel is too novel? Stream community thresholds at exceptionally low levels of catchment urbanization. Ecol Appl. 2011;21: 1659–78. 21830709 10.1890/10-1357.1

[59] Weijters M, Janse J, Alkemade R, Verhoeven J. Quantifying the effect of catchment land use and water nutrient concentrations on freshwater river and stream biodiversity. Aquat Conserv Mar Freshw Ecosyst. 2009;19: 104–112. 10.1002/aqc

